# The health workforce crisis in TB control: a report from high-burden countries

**DOI:** 10.1186/1478-4491-3-2

**Published:** 2005-02-24

**Authors:** José Figueroa-Munoz, Karen Palmer, Mario R Dal Poz, Leopold Blanc, Karin Bergström, Mario Raviglione

**Affiliations:** 1 Tuberculosis Strategy and Operations, Stop TB Department, World Health Organization, Geneva, Switzerland; 2 Department of Human Resources for Health, World Health Organization, Geneva, Switzerland

## Abstract

**Background:**

Human resources (HR) constraints have been reported as one of the main barriers to achieving the 2005 global tuberculosis (TB) control targets in 18 of the 22 TB high-burden countries (HBCs); consequently we try to assess the current HR available for TB control in HBCs.

**Methods:**

A standard questionnaire designed to collect information on staff numbers, skills, training activities and current staff shortages at different health service levels was sent to national TB control programme managers in all HBCs.

**Results:**

Nineteen HBCs (86%) replied, and 17 (77%) followed the questionnaire format to provide data. Complete information on staff numbers at all service levels was available from nine countries and data on skill levels and training were complete in six countries. Data showed considerable variations in staff numbers, proportions of trained staff, length of courses and quality of training activities. Eleven HBCs had developed training materials, many used implementation guidelines for training and only three used participatory educational methods. Two countries reported shortages of staff at district health facility level, whereas 14 reported shortages at central level. There was no apparent association between reported staff numbers (and skills) and the country's TB burden or current case detection rates (CDR).

**Conclusion:**

There were few readily available data on HR for TB control in HBCs, particularly in the larger ones. The great variations in staff numbers and the poor association between information on workforce, proportion of trained staff, and length and quality of courses suggested a lack of valid information and/or poor data reliability. There is urgent need to support HBCs to develop a comprehensive HR strategy involving short-term and long-term HR development plans and strengthening their HR planning and management capabilities.

## Background

The performance of health care systems is closely related to the numbers, distribution, knowledge, skills and motivation of its workforce, particularly of those individuals delivering the services [1]. Improvements in global health are greatly dependent on how well health systems can meet the demands placed on them by governments, programmes, communities and ultimately individuals. Human resources for health (HRH), all categories of clinical and non-clinical staff who make each individual and public health intervention happen, constitute a sine qua non of health systems. Therefore, developing HRH and fostering appropriate HR management are crucial steps towards achieving and sustaining improved and equitable health.

Tuberculosis (TB) constitutes the third most important cause of death and disability [2] among infectious diseases. It is estimated that in 2002, there were 8.8 million new TB cases worldwide (141/100 000) of which 3.9 million were sputum-smear positive (SS+). Despite control efforts, the global incidence of TB continues to grow in some regions, particularly in sub-Saharan Africa [3]. The emergence of AIDS and multidrug-resistant TB (MDR-TB) pose further challenges for global TB control; in 2000 there were 1.82 million TB-related deaths, of which 226 000 (12%) were attributable to HIV [2,4].

The World Health Assembly (WHA), in 1991, pledged countries to achieve detection of at least 70% of estimated infectious TB cases (SS+) and to cure 85% of them by the year 2000 [5]. Slow progress resulted in the deferral of these targets until 2005 [6]. Likewise, the United Nations commitment to sustaining development and eliminating poverty throughout the world led world leaders to formulate the eight Millennium Development Goals (MDGs), among them to halt, and begin to reverse, the global incidence of TB by 2015 [7]. The creation of the Stop TB Partnership in 1998 [8] and the Global Fund to fight AIDS, TB and Malaria (GFATM) in 2001 [9] represented significant developments in the fight against TB, thanks to increased financing and technical assistance made available to endemic countries. However, disease-specific programmes (including TB) are still struggling to meet their targets, and governments and their financial/technical partners have finally recognized this is largely due to shortcomings in the health care workforce [3,10].

Low-income and middle-income countries (LMIC) urgently need a sufficiently large health care staff with appropriate expertise, experience and motivation, working at the right places. Lack of HR strategies, inadequate HR planning and management, poor deployment practices, inflexible contracting arrangements and inability to create new posts or increase salaries resulting from international regulations capping social sector spending have contributed to the global HRH crisis [10]. In addition, poor salaries, low morale and worsening local economic circumstances result in low recruitment/retention, internal and external migration of trained staff and attrition of the health care workforce. The HIV/AIDS epidemic has increased pressure on health systems and causes death and disability of the workforce itself [11].

In 2003, national TB programme (NTP) managers from 18 of the 22 TB high-burden countries (HBCs, countries that together account for approximately 80% of the global TB burden), ranked inadequate HR first within the top five constraints to reaching the WHA global TB control targets [3]. Insufficient numbers, lack of adequately qualified or trained staff at different service levels, inadequate distribution, low motivation and poor staff retention were commonly described. Although some components of disease control programmes still remain vertical (e.g. drug procurement or laboratory QA) the majority of front-line services are now partially or totally integrated to primary health care and therefore a shortage of HR for TB control represents a shortage of HR for health delivery.

There is very limited published literature on HRH issues, particularly in LMIC and there are both scanty information on methods to assess HR capacity and lack of evidence on how best to evaluate interventions to strengthen and/or build HR capacity [12]. The dearth of published information probably stems from the persistent neglect of HRH development issues; in addition, research in HRH involves a broad scope of disciplines and often different research methodologies than those recognized in clinical medicine [13].

Currently there is increasing awareness that HRH concerns must be addressed in order to reach the MDGs, to expand access to priority interventions, to promote health systems development and to achieve global health equity [14,15]. However, without reasonably accurate information on numbers, location, qualifications and activities of staff, it is not possible to effectively manage or plan HR for the country's health services or for specific programmes [16].

This paper reports the results of a questionnaire sent to NTP staff in the 22 HBCs to assess the workforce available for TB control (staff numbers, cadres and skills), as well as the estimated HR requirements for appropriate TB control in HBCs. It aims to inform the development of more reliable methods of gathering qualitative and quantitative information on HR and to stimulate a long-overdue discussion on HR for TB control issues, so that governments of endemic countries and technical and financial partners can finally begin to address them jointly.

## Methods

### Participants

NTP managers and country-based WHO staff in the 22 HBCs: Afghanistan, Bangladesh, Brazil, Cambodia, China, Democratic Republic of the Congo, Ethiopia, India, Indonesia, Kenya, Mozambique, Myanmar, Nigeria, Pakistan, Philippines, the Russian Federation, South Africa, Tanzania, Thailand, Uganda, Viet Nam and Zimbabwe. These countries account for more than half of the world's population and approximately 80% of the global TB burden

### Questionnaire

Discussions with NTP managers and a literature review on HRH informed the development of a questionnaire to ascertain current staff provision; quality and intensity of training; time and type of personnel involved in performing different TB control activities; and estimated staffing needs at different health service levels. The questionnaire was pilot-tested internally within the Stop TB Department (STB), WHO headquarters, Geneva; and externally with staff from the NTP in Indonesia.

The first section of the questionnaire assessed staff numbers and skills. It included open questions about absolute numbers of staff involved in delivering TB control activities at each service level (provincial, district and health facility and laboratory personnel). Skills were assessed using a composite of the proportion of staff receiving training in the previous three years (at each level) and the quality of the training provided (length of courses, development of educational materials and/or use of standard -WHO or International Union Against Tuberculosis and Lung Diseases (IUATLD) – materials) as proxy measures.

Section two of the questionnaire addressed estimated HR gap. Since this paper specifically refers to TB control, we estimated the workload involved in adequate TB control in HBCs. Workloads were assessed by means of open questions on the different processes involved in the management of new SS+ patients (diagnosis and administration of a short course chemotherapy regimen under proper case management conditions including directly observed therapy, DOT), the estimated duration of each task and the type of staff involved in delivering them. A series of worksheets containing the different tasks required for TB case management were designed to assist respondents completing the questionnaire and to improve standardization. Capacity of health services at two different CDRs were assessed (current CDR and at the target 70% CDR). Workloads at current CDRs were calculated by multiplying the current numbers of new SS+ patients by the estimated time needed to treat a new SS+ patient; workloads at the 70% CDR were calculated by multiplying the figure corresponding to 70% of the estimated SS+ TB cases for each country by the time needed to treat a new SS+ patient. Eleven hours was used as the time needed to treat a new SS+ patient; this had been estimated previously by a TB experts' consensus (WHO, unpublished data).

### Survey

In March 2003, the questionnaire, together with detailed instructions in English, was e-mailed to NTP managers and country-based WHO staff in the 22 HBCs. Three e-mail reminders were sent monthly after the return deadline; follow-up with several countries included e-mail and telephone communications to clarify responses. Data were entered into spreadsheet software for the analysis; qualitative answers were discussed by the authors.

## Results

### Response rate

Nineteen of the 22 HBCs returned the questionnaire (86% response rate). Two countries provided information on their HR for TB control but did not use the questionnaire format and were thus excluded from the analysis; one country reported that it would take a substantial amount of time to get reliable information from such a large country and provided other readily available HR information; another reported that given the country's current staffing deficits and competing priorities they were unable to complete the survey. Further discussions revealed that some HR information was available in this country, although it was scattered in different sources and lengthy to compile. Both countries requested technical support to assess staffing needs at different levels and to assist their government's HR development programmes. Despite e-mail and telephone remainders, three countries failed to respond.

### Numbers of staff

Information on estimated numbers of staff was available from all 17 countries but complete in nine (53%) of them. Staff numbers within the same service level varied considerably between countries; i.e. numbers of staff at provincial level varied from 8704 in one country to 6 staff in two countries (Table 1); similar variations were seen at other service levels. More countries provided information on staff numbers at provincial than at district or health facility levels.; only nine countries (53%) provided numbers of staff at laboratory level.

**Table 1:** Staff numbers at each level and estimated numbers of trained staff in the previous three years (2000–2002)

**Country**	**Health service Level**							
	
	**Provincial**		**District**		**Health facility**		**Laboratory**	
	
	Total	Trained (%)	Total	Trained (%)	Total	Trained (%)	Total	Trained (%)
Afghanistan	40	22 (55)	360	-	-	-	-	30 (--)
Bangladesh	460	150 (33)	460	120 (26)	39329	750 (2)	1015	450 (44)
Brazil	27	19 (70)	-	20 (--)	-	6379 (--)	-	-
Cambodia	-	72 (--)	-	236 (--)	1120	705 (63)	-	-
DR Congo	56	19 (34)	306	-	4306	-	1000	-
Ethiopia	12	4 (33)	-	-	-	-	-	-
Indonesia*	70	70 (100)	420	420 (100)	1256	1256 (100)	405	405 (100)
Kenya	10^¶^	7 (70)	94	-	45900	1148 (2)	2121	350 (16)
Myanmar	-	8 (--)	-	276 (--)	-	18056 (--)	-	327 (--)
Nigeria	37	19 (51)	664	149 (22)	27000	295 (1)	3000	160 (5)
Pakistan	6	0 (0)	60	60 (100)	21000	500 (2)	600	200 (33)
Philippines	156	-	748	-	13900	-	2200	-
Russian Fed.^§^	-	-	-	4062 (--)	-	4197 (--)	-	1167 (--)
South Africa	9	9 (100)	-	(70–80)	202265	(70–80)	-	-
UR Tanzania	25	2 (8)	156	58 (37)	-	50 (--)	-	-
Uganda	6	3 (50)	55	-	1050	-	350	-
Viet Nam	8704	2205 (25)	2705	1248 (46)	10510	10510 (100)	804	624 (77)

* Percentage of training according to 2002 training plan

¶At regional level

§Federation

### Skills

Complete information on numbers of staff trained in the previous three years was available in only six countries (35%). There was also great variation in the numbers (or proportions) of trained staff (Table 1). Indonesia reported it trained 100% of staff at all levels, but this was based on its target for 2002; Viet Nam reported high levels of training at health facility and laboratory levels and South Africa reported high levels of training (70% to 80%) at provincial, district and health facility levels (although absolute numbers were missing). Other countries either reported very low levels of training or had missing information. Only six countries (35%) reported accurate information on training of laboratory staff.

Information on length of training courses and development of training materials was also incomplete. Length of courses for staff at the same service level varied greatly between countries; for example, courses for TB coordinators at provincial level (data from 11 countries) varied from 4 to up to 60 days, whereas at district level (information from 10 countries) courses varied from 4 days to 4 months (Table 2). Similar variations were reported at other levels. Development of training materials was reported by eight (47%); a further four (23%) reported developing lectures and exercises modules for training at all levels, whereas only three (18%) described using participatory educational methods. Nine countries (53%) had not developed specific training modules; five of them used WHO/IUATLD materials and the remaining countries used programme implementation manuals and guidelines as training tools. Altogether eight countries (47%) reported using WHO/IUATLD training materials. The WHO/STB training modules for health facility staff take normally five days to complete if all its tasks and units are included; only one of the countries that reported using it had a five-day course. Training at this level took three days or less in six of the 12 countries with available information.

**Table 2:** Length of training courses at different training levels and development of training materials by country

**Country**	**Length of training in days**				
	
	**TB coordinator**		**Staff health facility level**	**Laboratory staff**	Training materials*
				
	**Provincial level**	**District level**			
Afghanistan	10	-	-	7	No materials developed
Bangladesh	-	4–6	2–3	6	Developed lectures and exercises, also uses WHO materials for training at provincial and district levels
Brazil	5	5	5	5	Developed manuals & guidelines for all levels
Cambodia	-	5	3	-	Developed training modules at all levels
DR Congo	21	-	-	-	No materials developed, uses WHO materials at all levels
Ethiopia	-	7–10	5	5	Ad hoc handouts
Indonesia	12	12	6	8	Developed training modules for all levels
Kenya	15	-	2	3	No specific training materials developed, uses national guidelines and WHO & IUATLD training materials
Myanmar	5	5	1	5	TB manual for health facility staff and lab. technicians developed in 2002
Nigeria	21	120	3	6	Developed training materials at all levels, did not specify
Pakistan	-	10	variable^§^	10	Developed training modules, translated WHO materials for training lab. staff
Philippines	-	-	-	-	Use modified WHO materials at all levels
Russian Fed.	-	-	-	-	WHO materials were developed for Russia in 2002, use manuals and guidelines for staff at different levels
South Africa	4	-	3	-	No specific materials developed, uses WHO & IUATLD materials for different levels
UR Tanzania	14	30	5	5	Developed a TB Manual but no specific training materials
Uganda	15	-	-	-	Developed training materials but did not specify
Viet Nam	10 or 60	5 or 10	3 or 5	15–20	Uses national guidelines for training, for health facility staff it uses WHO & IUATLD training materials

* Development of specific training materials for each country or use of standard WHO / IUATLD modules and courses.

§Variable: Medical staff 6 days, paramedical 3 days, health workers 1 day.

### Performance

**Figure 1:**
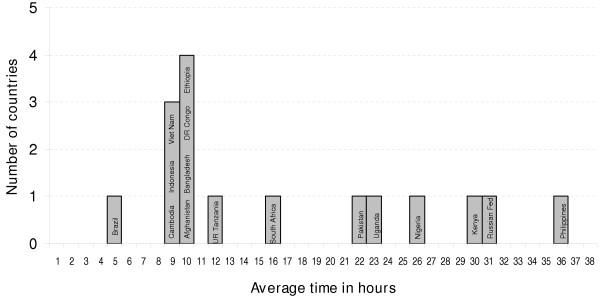
Average time spent to treat one new sputum-smear positive TB patient

The estimated time needed to treat a new SS+ patient is shown in Figure 1. Times were positively skewed and ranged from 5 to 36 (mode 10) hours. Eight countries (47%) needed from 9 to 12 hours; Brazil was the only country requiring less than 9 hours; Pakistan, Uganda, Nigeria, Kenya, Russia and Philippines were out-layers requiring between 22 to 36 hours. The Russian Federation (31 hours) and the Philippines (36 hours) took the longest, due to the policy to hospitalize all patients during the intensive phase of treatment in the former and the tendency to perform strict DOT during the continuation phase in the latter.

### Estimated HR gap

Sixteen countries (Table 3) reported information on estimated shortages of staff at peripheral level (health facility) at current or target CDRs. Two countries (12%) reported shortages of staff at current CDR, whereas five countries (29%) predicted health facility staff shortages at the 70% CDR. Estimated numbers of staff needed varied from 1009 more nurses in Afghanistan to 8981 health care workers in Myanmar; Uganda reported shortages of staff but did not estimate the numbers needed. Eleven countries (65%) reported no shortages of health facility staff at current or at 70% CDRs; these included countries with low current CDR and no data on staff numbers.

**Table 3:** Perceived staff needs at different service levels. NTP managers were asked to report perceived staff needs at different service levels. Staff needs at Health Facility level were evaluated both at current and at the 70% target case detection rates

**Country**	CDR*	**Perceived staff shortages**					**Comments**
			
		**Health facility**		**District**	**Provincial**	**Central**	
						
		**At current CDR**	**At 70% CDR**				
Afghanistan	19%^a^	No	1009	No	No	17	Poor distribution of staff, staff needed to run new facilities
Bangladesh	33%	No	No	No	No	1 – 5	
Brazil	84%	No	No	Yes			Capacity building of existing staff is a priority
Cambodia	52%^a^	No	No	No	No	No	Poor distribution and training of staff at district and central levels
DR Congo	52%^a^	No	No	No	52	>5	Capacity building of existing staff a priority
Ethiopia	33%^a^			Yes	5 (regional)	2	Lack of data, poor distribution and training
Indonesia	30%^a^	No	3670	Yes	Yes	Yes	
Kenya	49%^a^	No	No	No	No	8	Poor distribution of existing staff
Myanmar	73%^a^	Yes	8981	6	4	4	
Nigeria	14%	No	No	No	111	25	Capacity building of existing staff is a priority
Pakistan	13%	No	2981	Yes	Yes	>6	
Philippines	58%^a^	No	No	>3	Yes	>10	
Russian Fed.^§^	34%	No	No	No	No	Approx. 48	Not enough data available
South Africa	97%	No	No	Yes	Yes	Yes	Lack of funds for recruiting new staff
UR Tanzania	43%^a^	No	No	>364	>88	>11	Staff retention and deployment problems
Uganda	47%^a^	Yes	Yes	Yes (Zonal)		4	Inconsistent data provided
Viet Nam	82%^a^	No	No	No	No	No	Poor training of existing staff at district and provincial levels

* Case Detection Rate for 2002, Ref 3.

a No data available for the whole country; case detection rate for DOTS programmes

§Federation

All countries provided information on their estimated HR gap at district level; nine (53%) countries reported existing staff needs at district level (Table 3); nine countries reported existing needs at provincial level and all but two countries reported staff needs at central level. Some countries answered qualitatively while others estimated numbers needed, but it is not clear how these estimates were reached. Cambodia and Viet Nam were the only HBCs reporting no staff shortages at any service level. Afghanistan, Cambodia, Ethiopia, Kenya and Uganda reported poor distribution of staff. Brazil, Cambodia, Democratic Republic of the Congo, Ethiopia, Nigeria and Viet Nam reported training and capacity-building shortfalls.

## Discussion

In many HBCs, NTP managers do not have access to accurate information on numbers, types and distribution of staff involved in TB control activities. As previously stated, TB control programmes are partially or fully integrated into health care systems; this lack of HR information thus could at best indicate poor communication and coordination between NTP managers and HR planners or, at worst, a general lack of information on HR in the health care system. In order to adequately manage and plan HR it is important to have up-to-date information on the quantity, distribution and skills of the existing health care workforce [16]. It is therefore imperative to assist HBCs to develop and maintain appropriate HR databases so that the necessary information for planning and managing their health care workforce can be readily accessible.

Accreditation is often used as a proxy for competence for professional groups (doctors and nurses); for other health care staff, adequate competence is ensured through regular training and supervision. Apart from Indonesia and Viet Nam, there was no correlation between information on numbers of staff, attendance of training courses, length of courses and development of training materials. In general NTP managers in HBCs had limited information on staff attendance of training courses and on the characteristics, duration and intensity of training activities. HBCs need to develop needs-based comprehensive training policies and training strategies for health care staff at all service levels, incorporating pre-service training, re-training, in-service support and continuous professional/career development. Similarly, it is important to emphasize the role of periodic monitoring and supervisory visits as part of the staff continuous education and support processes. Appropriately designed strategies fostering career paths could increase staff motivation and performance; improve staff recruitment, retention and distribution; and even have a positive effect on enlisting of students into training programmes [17] and expansion of the health care workforce.

The quality of training materials was also of concern: many countries used inadequate training tools, few had developed specific modules and even fewer used problem-based learning or participatory methodology to facilitate adequate skill development. The length of courses also varied greatly: while very short courses do not allow the development of skills and competences needed to improve performance, lengthy courses have economic and logistic implications. Differences in the quality and intensity of training could translate into service or performance variations with detrimental effects to programmes and ultimately to patients. There is a need to standardize training in terms of contents, competences, methodologies, course duration and quality of training materials. Improvement and standardization of training curricula and methodologies will facilitate the adoption of a universal standard of care for TB patients in HBCs, leading to a more rational use of the health care workforce, improved staff motivation and productivity and better outcomes for TB patients.

Although the majority of countries described deficiencies of staff at central level, few reported shortages in the actual numbers of posts. However, there were concerns about the distribution and/or the skill mix (competences and efficiency) of staff. There was no clear relation between reported data on staff needs and either the TB burden in the country or the actual performance of the NTP. It is of concern that only Uganda and Myanmar reported shortages of staff at current CDRs, whereas countries with very low current CDRs did not. Only five countries projected shortages of staff at the 70% CDR; four of them estimated large numbers of staff required.

If the assessment of the HR needs was based on the actual workload, it is possible no substantial gap was determined because most of the current diagnostic and treatment tasks are carried out satisfactorily by existing staff, but an unrecognized HR gap could hamper improvement in case detection rates. The inability of some countries to increase the number of posts because of recruitment ceilings imposed under structural adjustment programmes could lead to countries underreporting their HR gap. Further analysis and recommendations on staff needs at different levels is hindered by the lack of accurate information on numbers, quality and distribution of existing staff.

This survey is, to our knowledge, the first attempt to ascertain HR for TB control in HBCs. The response rate was high and responses were analysed in the context of the countries' estimated TB burden and current CDRs.

Limitations of the study are evidenced by the great variation in reported staff numbers and the lack of correlation between information on numbers of trained staff, length of courses, development of materials and performance, suggesting a shortage of valid information, poor standardization and/or data reliability.

The fact that the questionnaire was in English could have generated some inaccurate answers. Indonesia, for example, reported 100% training completion based on its 2002 training goals rather than in absolute numbers (the actual proportion of staff trained at health centre level was 35%), but countries were re-contacted (by e-mail or telephone) to clarify inconsistent replies. Responses could have been biased towards greater HR needs if countries perceived this as an opportunity to request increased support; however, this did not seem to happen, since few countries reported staff shortages.

The performance assessment component of the questionnaire assumed NTP managers could assess the HR gap by comparing existing staff numbers (at different service levels) with the product of the additional SS+ cases by the time spent in diagnostic/treatment activities. Although all the tasks were listed in the questionnaire, the interpretation of what constituted achieving each task was left to NTP managers; this could have affected their estimation of the time required. We are currently developing a more detailed task analysis tool that includes the breakdown of each TB control activity into specific single tasks and a description of what constitutes each task; this will facilitate future studies. Furthermore, this method accounted only for new SS+ cases: in some countries, health facility staff will spend more time dealing with relapses, treatment failures or other TB patients: On the other hand, in some HBCs some tasks are performed by NGOs or the private sector and this was not discounted.

Constraints to HR development such as poor HR data quality, lack of a comprehensive national HR plan/strategy and little attention to continuing education programmes [18] were common to HBCs. This study evidences the huge gap in HR data (more apparent in large countries such as China and India) and the variability in quality and validity of available information. This is not an isolated problem of TB control programmes, given the dearth of published data on HRH in LMICs. Decentralization, ongoing in many HBCs, could have contributed to worsening HR information at central level. Developing HR planning and management capacity at district level and generating HR information systems should accompany decentralization processes [19-21] so that decentralization does not result in decentralized chaos [19]. A comprehensive HRH database facilitates HR surveillance and the management and planning of HR development in the health system [20].

The 2nd ad hoc committee on the TB epidemic recommended addressing the health-workforce crisis by collaboratively developing policies to reduce barriers to creating and filling posts in HBCs; increasing staff recruitment and retention by improving working conditions in the health sector; promoting task analysis and HR needs assessments, HR planning and training at country level; and working together with other stakeholders to develop strategies to further mobilize HR for TB control [22]. In general there is poor communication between HR planning units in the MoH and other technical programmes. A starting point for many HBCs will be a rigorous appraisal of their current HRH through in-depth assessments. Calculating programme-specific HR requirements, and informing the HR planners in the MoH, are two steps that are not consistently performed, resulting in a health care workforce often unaware of its own capacities and limitations.

## Conclusion

(See Table 4.) There is urgent need to assist HBCs developing HR information systems so that the up-to-date information required for appropriate planning, managing and supporting their health care workforce can be available. NTP managers in HBCs were generally aware of the need for appropriately trained staff at different service levels and, in some countries, the need to redistribute existing staff. Many HBCs require support in developing HR planning and management capabilities; however, more information is needed from countries to understand what factors most influence HR capacity so that country-specific plans can be developed.

**Table 4:** Conclusions

1. A paradigm shift in our approach to HR is needed. The HR impact of health initiatives must be conveyed in an explicit, open and unambiguous way so that governments, planners, and financial and technical partners will have a clearer understanding of the urgency of the HR crisis and will have to take a stand on addressing it.
2. HR information systems in HBCs must be developed/strengthened. Without some reasonably accurate information on the numbers, location, qualifications and skills of staff it is impossible to administer, manage or plan the health workforce in any effective manner.
3. There is a dearth of information on HR for disease control programmes in LIMC. There is a need to develop HR assessment tools allowing for the different disciplines involved in HR issues and to conduct in-depth studies using validated methodology.
4. It is important to improve the communication link between technical programmes and HR planning at central level. There is a need to support some HBCs in developing HR management skills and in capacitating personnel in the area of HR management and planning.
5. Training is an important component of HRH development; there is a need to identify the minimum requirements of training at different service levels required to obtain a universal standard of care for TB patients and to better standardize training materials, methodologies and courses.
6. There must be a twin-track approach to addressing the HRH crisis. Current shortages must be addressed with short-term interventions in line with medium/long-term solutions developed within the context of poverty reduction strategies and national medium-term expenditure frameworks.

Human resources constraints in TB will not be solved by NTPs in isolation; they are and will remain a subset of the general health workforce. The degree of integration of the HR for TB within the health workforce will vary from partial to total, depending on local conditions. The same holds true for the supply side: different health programmes compete for finite HR, posing a strain on local health systems. The health workforce crisis for TB control must be addressed within the broader HR context. Without creative solutions there will not be enough trained health professionals to implement the strategies proposed by the priority disease control programmes.

Finally, a paradigm shift in the way we approach HR issues is needed. Up until now, the HR implications of public health interventions have been tacitly understood; the impact of healthcare initiatives on the limited health workforce has always been implicit, unspoken and often underrated. There is now a need to clearly convey the HR impact of health initiatives in an open, unambiguous manner. By endeavouring to make the HR implications of each existing and new public health intervention explicit, we hope governments, planners, and financial and technical partners will have a clearer understanding of the urgency of the HR situation and will have to take a stand on addressing the health care workforce crisis.

## Conflict of interests

The author(s) declare that they have no competing interests.
